# Domestic violence in Mozambique: from policy to practice

**DOI:** 10.1186/s12889-021-10820-x

**Published:** 2021-04-22

**Authors:** Eunice Jethá, Ines Keygnaert, Emilia Martins, Mohsin Sidat, Kristien Roelens

**Affiliations:** 1 Community Health Department Eduardo Mondlane University, Faculty of Medicine, Salvador Allende Avenue, 702 Maputo, Mozambique; 2 International Centre for Reproductive Health, Faculty of Medicine & Health Sciences, Ghent University, Ghent, Belgium; 3 MIHER Mozambican Institute for Health and Research, Maputo, Mozambique; 4 Department of Obstetrics & Gynaecology, Faculty of Medicine & Health Sciences, Ghent University, Ghent University Hospital, Ghent, Belgium

**Keywords:** Domestic violence, Care provision, Care providers

## Abstract

**Background:**

To reduce the impact of domestic violence (DV), Mozambican governmental and non-governmental entities are making efforts to strengthen the legislative framework and to improve the accessibility of care services for survivors of violence. Despite this remarkable commitment, the translation of policies and legislation into actions remains a considerable challenge. Therefore, this paper aims to identify gaps in the implementation of existing national policies and laws for DV in the services providing care for survivors of DV.

**Methods:**

This qualitative study comprised of two approaches. The first consisted of content analysis of guidelines and protocols for DV care provision. The second consisted of in-depth interviews with institutional gender focal points (Professionals with experience in dealing with aspects related to DV). The analysis of the document content was based on a framework developed according to key elements recommended by international agencies (PAHO and UN) for design of DV policies and strategies. Data from the in-depth interviews, where analysed in accordance with the study objectives.

**Results:**

Eleven (11) guidelines/protocols of care provision and innumerable brochures and pamphlets were identified and analysed. There is a standardised form which contains fields for police and the health sector staff to complete, but not for Civil Society Organisations. However, there is no specific national DV database. Although the seventeen (17) focal points interviewed recognised the relevance of the reviewed documents, many identified gaps in their implementation. This was related to the weaknesses of the offender’s penalisation and to the scarcity of care providers who often lack appropriate training. The focal points also recognised their performance is negatively influenced by socio-cultural factors.

**Conclusion:**

Within services providing care to survivors of DV, a scarcity of guidelines and protocols exist, compromising the quality and standardisation of care. The existence of guidelines and protocols was regarded as a strength, however its implementation is still problematic. There was also recognition for the need to strengthening by governmental and non-governmental entities the defined policies and strategies for DV prevention and control into practice.

## Background

Domestic violence (DV) is recognised as a complex problem affecting a large proportion worldwide, violating their human rights [1–3]. DV is often defined as an aggression, abuse or threatening behavior between intimate partners or former partners and members of a family occurring in the household [4, 5]. Due to the fact women are often described as survivors, the term DV is often replaced by intimate partner violence, violence against women, spousal or ex spousal violence, family violence and wife assault or abuse. Although men are identified as the most frequent perpetrators, women can also perpetrate DV against their male partners, however with some differences regarding, backgroud causes/motivations, treatment/monitoring and penalties in male to female and female to men partners violence [6–11]. Although this type of violence is often described as being unidirectional (male-female or female-male), it must be recognised it can also be bidirectional or mutual. This is when the man or woman may act as perpetrators and victims in different situations. Mutual partner violence has as frequent predictors exposure to childhood abuse and violence between parents [12, 13].

Worldwide, one in three women has been physically and/or sexually abused by her intimate partner or family members at some point of her lifetime, compromising their physical, mental and reproductive health. According to a World Health Organisation (WHO) multi-country study, the prevalence of women survivors of physical or sexual abuse from their intimate partner ranged from 15% (Japan) and 71% (Ethiopia) [14–18]. The low rate of victimisation experienced by men in relation to women is often described as not always due to gender difference. Rather the incongruity with which this phenomenon is addressed plus the influence of socio-cultural factors places men in an extremely awkward position in society. Some studies reveal approximately 38.9% of men have suffered sexual victimisation at some point in their lives (just one of the ways in which DV can manifest) [19].

Similarly, compared to the rest of the world, Sub-Saharan African women are more affected by DV than men. The two most common forms of violence against women in the domestic sphere in some Sub-Saharan Africa countries are verbal and physical violence, which are attributed to poverty, as well as to contextual and cultural factors [20, 21].

Mozambique is not an exception, according to the Demographic Health Survey (DHS) of 2011, one-third of women (33%) have been victims of physical violence since the age of 15, at some point in their lives in a domestic environment. Most recently, Malaria, HIV/AIDS, and Immunisation Indicator Survey in Mozambique (IMASIDA) found approximately 24% of the women interviewed over the age of 15 admitted to being victims of DV, more specifically physical (18%), emotional (15%) or sexual (3%), at some point of their lives. Although less frequent, approximately 13% of men reported having been survivors of DV, with the intimate partner as perpetrator [22, 23].

Hence, in Mozambique, the rise of awareness of DV has led the government to place this phenomenon as a priority on its political agenda attempting to reduce cases of DV and to improve the quality of survivors’ lives. To respond to the magnitude of this problem, governmental and non-governmental entities are making efforts to strengthen legislative framework and to improve the accessibility of care services for survivors of violence. As a result of this effort, Mozambique has a wide range of legislative measures, produced and approved by the Mozambican government.^1^

Apart from this political commitment, identification of the key role institutions for DV-related issues – the Ministry of Gender Child and Social Action, Ministry of the Home affairs, Ministry of Health and Justice as well as the establishment of a network between governmental and/or non-governmental organisations were the other fundamental aspects taken into account in the design of the Mozambican legal structure for DV prevention, and importantly, control of its implementation [24, 25]. In the context of the institutional network, integrated care for survivors of DV (involving a multisectoral approach) and the identification of gender focal points at the level of each institution was recommended. Gender focal points are professionals who are supposed to have experience in dealing with DV-related issues and are appointed to ensure an integrated approach, through the implementation of programs and guidelines in the management of DV cases [24]. An integrated approach is understood as a comprehensive and coordinated response involving governmental and non-governmental entities [26, 27]. To complement these efforts, the government has created DV survivor’s assistance offices attached to the police stations in all provincial capitals.

Despite these remarkable efforts, cases of violence are still underreported and neglected [20, 28, 29]. One factor that could be behind this phenomenon, is the recognition of DV as a private matter, with only 10% of all cases of violence being reported to the police. Most often, survivors appeal to the informal help system (community leaders, extended family) to solve the problem [25, 30]. The lack of information on the availability of services and poor quality of services partially explain low reporting and also minimal care-seeking behaviour of DV survivors or their relatives [17, 31, 32].

Furthermore, policy and legislation translation into actions through development of guidelines or service delivery protocols as well as implementers or care providers commitment remains an enormous challenge [33, 34].

Therefore, this paper aims to identify gaps in the implementation of existing national policies and laws for DV, developed at the central level namely the Ministries of Health, Home affairs, Gender, Children and Social Action, and Civil Society Organisations. In addition, for the purpose of the study, institutions of the implementation level were also included, i.e., those who provide care to survivors of DV. More specifically, at care provision sites, we first verified the existence of guidelines and protocols on care for DV survivors. Secondly, we analysed the extent to which guidelines and protocols reflected recommendations established in law, policies and strategic plans. Thirdly, we assessed gender institutional focal points’ awareness of national policies, laws and strategic plans on DV. Finally, we describe the gender institutional focal points’ perception of primary gaps in the implementation of policies, laws and strategic plans regarding DV.

## Methods

### Study sites

This study was conducted in two places, namely in Maputo City, the capital of Mozambique, located in the Southern part of the country and in Quelimane city, the capital of Zambézia province, located in the centre of Mozambique. The reasons for choosing these cities were the following. According to the last DHS, in Zambézia, 30% of women were survivors of DV, and 70% of them revealed they have never asked for help, nor ever told anyone about the incident of violence. In Maputo City where supposedly all care services are based and where probably of access to information is higher, the prevalence of DV was 38% never asked for help and 42.8% never told anyone about episodes of violence [22].

The study included institutions, purposely identified due to their responsibility in addressing DV-related issues. Therefore, in Maputo City Ministry of Gender Child and Social Action, Ministry of Health, Ministry of Home Affairs as well as other governmental institutions at the central level were included. Whereas in Quelimane City, the Provincial Directorate of Gender, Social Action of Zambézia; and the Provincial Directorate of Health of Zambézia^2^ were selected [24].

**Fig. 1:**
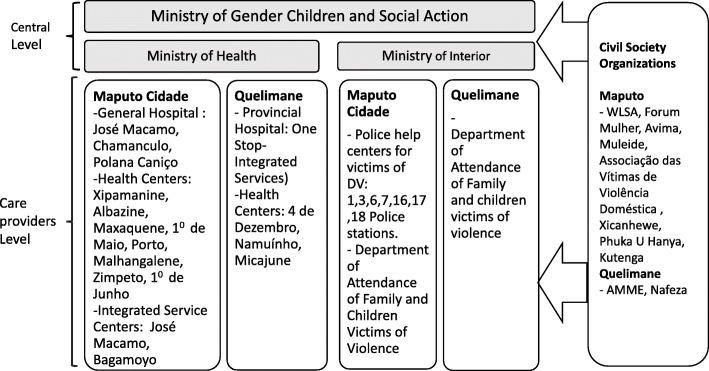
Institutions included in the study

In addition to these institutions at central level of the Mozambican government, r relevant institutions at care providers’ level were visited (see Fig. 1): Health units of primary, secondary and tertiary level, police stations, departments of attendance of family and children victims of violence, and Civil Society Organisations (CSOs). CSOs are responsible for community-based activities and for social support for DV survivors (the link between community and other care services, such as advocacy, provision of shelter for DV survivors) and collaborate with institutions part of the multisectoral mechanism on survivor’s care provision [24, 25, 35]. For this study we will consider CSOs as a non-governmental and non-profit entities that do not represent the governmental entities. CSOs include formal and informal organisations that act within one thematic area (DV-related issues) or in multiple areas (HIV/AIDS, child marriage, among others) of interest or concern of the society [36].

### Study procedures

This study used a qualitative approach for the analysis of the documents and of in-depth interviews with institutional gender focal points. The study was conducted between March and June 2017.

**Fig. 2:**
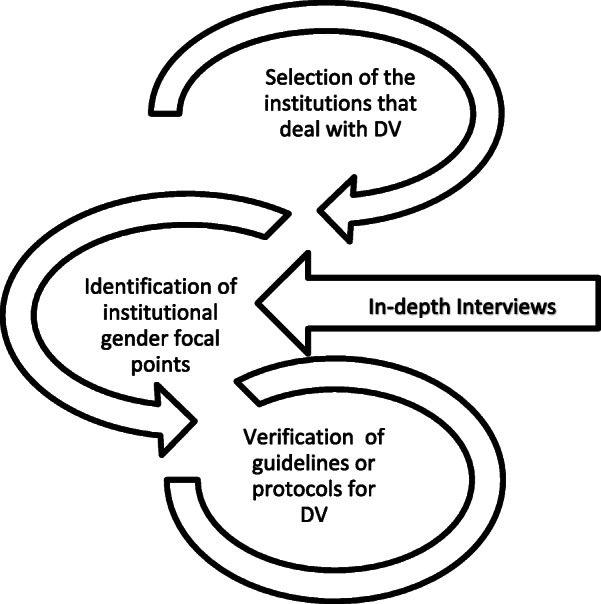
Study data collection procedures

To conduct this study the following steps shown in the figure below (Fig. 2).

Document analysis was based on verifying if the guidelines or protocols reflected the recommendations drawn in the national policies, legislation and strategic action regarding institutional plans.

To facilitate analysis guidelines and protocols, the contents of components recommended by the Pan-American Health Organisation (PAHO) and the United Nations (UN) relating to the design of policies and strategies for DV management were used. These components included document title (naming title), the inclusion of the DV definition, the main strategies especially the integrated approach and the beneficiaries of these documents [37, 38].

**Fig. 3:**
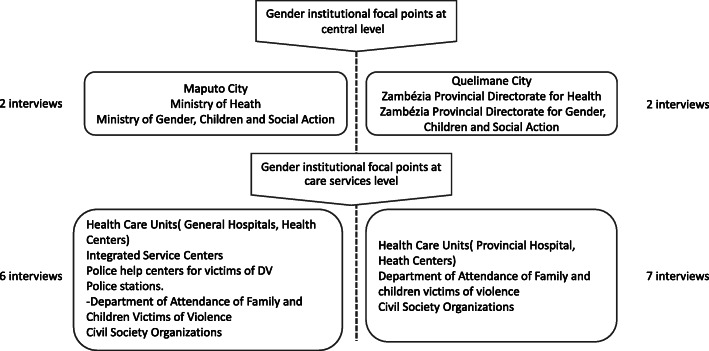
In depth-interviews with gender focal points at different levels of care in Maputo and Quelimane cities

In addition to the document analysis in-depth interviews were conducted as shown in Fig. 3. An interview guide was developed by the principal investigator and reviewed by the other investigators considering the study objectives. It was tested with professionals providing care to the victims of sexual violence at the Central Hospital in Maputo (two forensic doctors, one gynaecologist and one paediatric surgeon). After piloting, the interview guide was reviewed and adopted, with minor modifications, for use in this study. To facilitate data collection as well as analysis of information, interviews were recorded and subsequently transcribed, assuring confidentiality and anonymity.

All guidelines and protocols of attendance for the care of DV survivors, referred by gender focal points at the governmental central level (Fig. 3) were included in the analyses. At the level of care services for DV survivors, all existing guidelines and protocols (physical copies) to provide care to survivors of DV were analysed.

All documents that were not cited by the focal points or were not designed specifically to provide care to DV survivors, were excluded. At the care services, we also excluded all documents that although referred to by gender focal points, were not physically available at these locations.

### Selection of the sample

Qualitative document analysis – Data were collected at specific institutions randomly selected using 50% of all existing care services, based on a sampling program that generates randomness, [39] (See Fig. 1). A total of thirty-eight (38) care services for survivors of DV were included in the study. Of these, eighteen (18) belong to the Ministry of Health, nine (9) to the Ministry of home affairs, and eleven (11) are CSOs. Specifically, selected institutions included: three (3) general hospitals, two (2) health centres, two (2) integrated service centres, seven (7) police help centres, two (2) Department of attendance for family and children victims of violence and eleven (11) CSOs.

Qualitative in-depth interviews – To substantiate results obtained in the document analysis seventeen (17) gender focal points of each institution under study (see Fig. 3) were purposively identified by the responsibility of the institution and then they were invited to participate in the study. Focal points specifically included were police officers, health care professionals, social assistants and policy makers at the central level, were informed of the study aims by the investigator and field assistant.

All focal points identified had shown their interest in participating in the study, and an appointment was scheduled according to their availability, for interview and verification of guidelines and protocols for care provided to DV survivors.

Interviews were conducted by the principal investigator or a trained assistant, in Portuguese, the official language of Mozambique. This language was also spoken by all gender focal points.

### Data analysis

We selected, verified, and screened policies, laws, and institutional strategic/action plans on DV by analysing the documents. According to their origin and type in conventions treaties, documents were listed. These included declarations (international documents) policies, laws, strategic, and action plans (national-Mozambican documents). As well, adoption dates and final approval were taken into consideration. For document content analysis, critical cartography was conducted. For this study, cartography refers to identifying, compiling, and mapping all DV-related documents under examination. This critical mapping is accomplished by considering the type of paper analysed and its content. This ensures a synthesised illustration of all included documents [40]. Documents were listed according to their kind in guidelines/protocols, brochures as well as pamphlets. Analysis of the report under study was based upon the Walt and Gilson model to analyse health policies. It employs the triangulation of context in which the policy is developed. Its contents, as well as its main actors involved in the process of policy design and implementation, are examined [41]. In this specific study, although we have used the model described above, we will only report on the analysis of its contents, not policy context. To the focal points, specifically implementers and policymakers, an in-depth interview was conducted (described below). To standardise this process specific groups were created taking into account each element of the framework mentioned above. Thus, for the naming style, documents such as “Violence Against women”, “Violence”, “Gender equality”, “Family” and “Domestic Violence” were considered for analysis. Beneficiaries considered were: “women”, “child”, “family” and “population in general”. Although “women”, including young girls and children also part of the “family”, were disaggregate in two groups given their specificities regarding DV. Prevention, Assistance, DV notification, Advocacy, Capacity Building, Monitoring & Evaluation, Protection and Offender Criminalization were analysed as other strategies.

The various forms of DV, more specifically physical, psychological, sexual and economic violence were taken into consideration. Moreover, “human rights” was also included since DV is defined as a violation of human rights. The catch-all category of “other”, consists of further forms of DV, such as female genital mutilation, incest, premature marriages,^3^ trafficking women and moral violence. After this process of systematising all analysed documents, it was verified as to what extent its content is aligned with national laws, policies and strategic plans dealing with DV. For this alignment, the same analysis previously made in the laws, policies and strategic procedures were used as a basis.

**Fig. 4:**
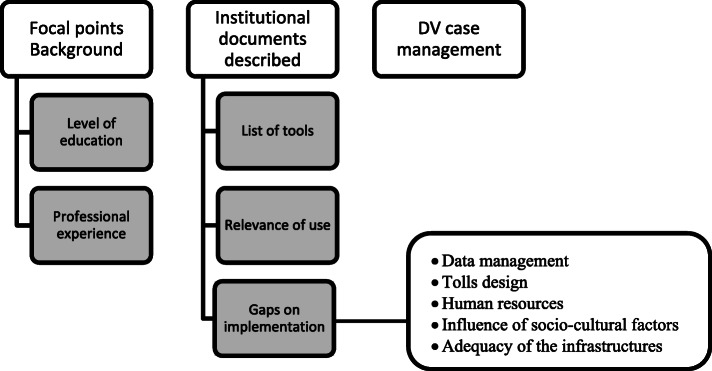
Nodes and sub-nodes used to perform the data analysis

After conducting in-depth interviews, a deductive content analysis was performed. This transpired in several steps to obtain critical themes emerging from the data [43]. Firstly, a categorisation matrix was created to consider study objectives, and previous knowledge was further developed. Secondly, audio-recorded interviews were transcribed in Portuguese. Transcriptions were read thoroughly by (EJ) one of the authors to achieve a sense of the whole and to ensure accuracy, discussions with the researchers’ uncertain meaning. The next step was the revisions of the transcripts and discussions by the research team to ensure the information was as refined as possible. Lastly, the transcripts were exported to NVivo version 12 (QSR International Pty Ltd., Doncaster, Australia) and all transcripts were coded with the initial categorisation matrix. Sub-themes emerged within these categories as coding progressed and were incorporated to the matrix, according to Fig. 4 [44, 45].

## Results

This section gives detailed results of the document analysis and in-depth interviews. In line with the study objectives, in the document analysis, we will report on the type of document analysed and its alignment with national policies. Comprehensive interviews describe the relevance, gaps and constraints of protocols and guidelines under study. These are taken from the point of view of gender focal points and care provision guidelines/protocols.

### Document analysis

This study includes approximately 11 guidelines/protocols and many brochures as well as pamphlets, (see Table 1). These documents were designed at the central governmental level to be implemented at the care services level.

**Table 1:** Guidelines or protocols included in the document review

**Institutions** General hospitals; Health centres; Integrated service centres	**Guidelines** Management of child victims of sexual violence Management of sexual violence Guide for Integrated Care for Victims of Violence Performance measurement of the tools of Integrated care for victims of Gender-based Violence Gender-based Violence Attendance **Flowcharts/Forms/Reports/Other** Violence case notification form. This includes sexual, physical and psychological violence. Record book.
Police help centres; Department of attendance for family and children who are victims of violence	**Guidelines/ Forms/ Others** Procedures for Assistance to Victims of Domestic Violence by Firearms or the White Weapons Program of care for women and children victims of violence Referral of Violence Cases Violence Case notification book Flowchart of care for victims of violence **Information, Education and Communication Material** Brochures, Pamphlets.
Civil Society Organisations	**Information, Education and Communication Material** Brochures, Pamphlets

However, as we went down to the level of care services, a marked decrease in the availability and awareness of the existence of guidelines, protocols, brochures and pamphlets was noted.

#### Alignment of documents with national policies

##### Naming style

Given the use of different themes in analysed documents, the non-standardisation of titles is noteworthy. In the care services for survivors of violence under the responsibility of the ministries of health and home affairs, the most frequent themes found in the title of the reviewed documents were violence in general, gender-based violence and sexual violence. In relation to the CSOs, titles of pamphlets, brochures and strategic plans contained themes encompassing varied problems – DV, sexual violence, violence against the elderly, and trafficking of children.

##### Definition of domestic violence

The relevance of including the definition of various forms of violence in guiding documents for the provision of care to survivors of violence is recognised. However, the majority of reviewed documents do not have the definition in their content. This definition of numerous forms of violence was only included in some IEC materials disseminated by CSOs.

#### Main strategies

##### Integrated approach

Guidelines and protocols verified in the health and home affairs institutions are aimed to provide assistance to DV survivors. The violence case notification form is a standardised tool that integrates multiple DV-related issues. This tool was developed through the engagement of multiple sectors, civil society advocates and survivors of DV. In this form, health providers and police have a specific role to play with the survivor including taking full information, and the procedures completed are specifically focused on care provision. Although CSOs have been working with the communities, identifying many cases of DV, they were not included in the violence case notification form, thus leaving them out of the integrated approach.

In health centres and general hospitals, we did not find guidelines or protocols mentioning integrated care. The existing documents referred solely to sexual violence in children and adults, more specifically after exposure to HIV/AIDS and are part of a project funded by a Non-Governmental Organisation, Pathfinder International.

##### Other strategies

Regarding DV case notification, although there is a standardised form issued by the government, health and home affairs institutions have their own case registration book. Advocacy is the main activity of the CSOs not presented in the reviewed documents from other institutions under study. Although capacity building was a daily activity of the institutions under study, only the CSOs have the document that supports the implementation of this activity.

The Ministry of health was the only institution at the central level to present an instrument for monitoring and evaluation of DV-related activities. Conversely, the same instrument was not utilised at health facilities (implementation level).

##### Beneficiaries

Almost all documents analysed had the general population as beneficiaries. Only one, on sexual violence, indicated the child as the main beneficiary. Since the majority of the CSOs have their pillar based on the human rights of women and children, some of the pamphlets did address aspects related to these specific groups.

### In-depth interviews

#### Relevance of guidelines and protocols

Aproximately half of the interviewees emphasised the relevance of the documents under analysis. All were unanimous in stating these were crucial to guiding them through the steps to be taken in the management of DV cases.

*“The operational plans assist in the implementation of the actions of all stakeholders ... all sectors of both government and civil society are urged to take action in this area based on these instruments”, FP_01_GCSAM.*

*“We actually have everything, within the health facilities, protocols on what you should do. Protocols, for example, for sexual violence management of cases of, what you should do to a victim”, FP_02_HM.*

In addition to strengthening the care of survivors of violence, the documents under analysis have been described as crucial in publicising laws and raising awareness about DV-related issues.

*“. ...we took the law, divided it and made brochures with very simple language. So that even people without legal training can understand and reproduce the law … So, we take the results of that research and we transform it into various formats to reach many different target groups ok, FP_01_CSO.*

#### Guidelines, protocols, gaps and constraints

Although the relevance of documents under review has been recognised by some focal points, most of them cited gaps in the implementation of these protocols and/or flowcharts.These gaps vary from those related to the documents themselves. For example, gaps related to policies, content, and more specifically, the inclusion or non-inclusion of a DV definition, beneficiaries and main strategies. Besides this gap, there are other related inconsistencies in the process of implementation. These were also described.

##### Policy content

Although this point has been raised a by few focal points (FP_01_CSO, FP_03_CSO and FP_03_CSO). These focal points recognise the law on DV is not perfect. One glaring constraint related to the design of policies is the penalisation of perpetrators. According to existing focal points, DV is considered a crime. However, some articles of the criminal code do not refer to the penalisation of the perpetrator.

*“For example, in the law of violence you have a situation of an article that refers for example to the penal code for this penal code already reviewed, but nevertheless does not tell you which article. Refer to the penal code that has several articles. This is a gap....”, FP_01_CSO.*

Moreover, the penal code is unclear regarding crime concealment, where family members up to third grade are not considered cover-up being exempt from penalty.

“*… .the crime of concealment is penalised. But this article says the following: ...they don’t fit into this category; they can’t be penalised for these aspects, family members up to third degree”, FP_03_CSO.*

Another constraint is the absence of an appropriate field in the standardised form for the registration of procedures carried out by CSOs in DV case management.

*“....where do we fit in? ....... What support has this victim had? For example, where was she/he referred to, then from here where she/he was referred to … and how it was, what was the outcome of this case”, FP_05_CSO.*

##### Policy implementers

In addition, gaps related to policy implementation, such as the small number of care providers for DV survivors, lack of personnel training of DV, and their attitudes and practices were described by gender focal points at the central and implementation level. Issues related to the context in which the implementation of the policy is framed, were also raised by the focal points.

*“Some units already have a psychologist; we still do not have them here. Maybe in this part, cases... we can even refer... in the district direction level, we have a psychologist, so we can be referring there”, FP_06_HM.*

Othere mentioned aspects are related to the lack of training. This compromises the performance of care providers in the identification of probable survivors of DV and no less importantly, how to manage DV cases.

*“...You look and say you slipped with the basin and got the black eye, right? And a good diagnosis is not made to certify if in fact she/he in fact slipped in the stairs and we end up registering with other diagnosis and not the domestic violence. So, there’s a lot of work... a lot of work...”, FP_01_CSO*.

*“...So, if you, a care provider are not properly trained, you will not be able to get the victim to open up to you. You will think that she is there in the health unit because she has another concern while she does not. She needs your support much more” FP_02_HM.*

Besides the facts mentioned above, most gender focal points inferred that providers’ attitudes and practices can also negatively influence their performance as well as compromise the implementation of policies, laws and strategies to prevent and control DV.

*“… The big gap is in the health care provider itself. It’s sad to say, but we do not have the habit of reading. We do not read, we have the documents there and we do not read and we keep doing …*.” *FP_02_HM.*

*“… some magistrates who simply do not want to apply the law of violence, although they are ready, too much work being done we still continue to have this question” FP_01_CSO.*

*“And if the nurse or the technician does not refer her to the office of attendance, to the office of attendance at the district-level, this case is likely to be lost.” FP_01_IM.*

##### Policy implementation

During interviews with gender focal points, they cited the influence of socio-cultural factors as a barrier to the implementation of policies, laws and strategies to eradicate DV. In addition to the financial status of the victim, most of the focal points pointed to community attitudes and practices in general and more specifically, of the survivors themselves as potential barriers to achieve the expected results.

*“But I realised that for example in the peripheral areas I think that poverty influences a lot. Someone who sees that he is mistreating me but gives me bread. And if I leave him, where will I have bread? Where will I have something to wear and where I will have a place to sleep”FP_01_IM.*

*“We know that DV is a public crime and that often people do not take it seriously or in consideration... This is their house’s problem. It’s their home issues … but the person is dying, and they leave children. These children are in responsibility of the government … These are children who need assistance” FP_02_GCSAM.*

*“At the entrance door... we do medical care, right? Then comes the legal part, but unfortunately most of our victims do not complaint”,FP_01_HM.*

*“I’ll complain to my husband and then I’ll come back. I share the bed with him. I’ll complain to my husband then I’ll come back and share the table with him. I think there is one here... we must overcome this taboo and let people free themselves and start to live life”, FP_01_IM.*

## Discussion

### Documents alignment with national policies

That political and institutional sectors have to control and prevent DV is evident from the number of existing guidelines, protocols, pamphlets and brochures developed. Although they might exist, their implementation still needs to improve services providing care. The lack of guidelines at care services sites can be linked to the lack of financial and human resources suitably qualified and committed to addressing the DV-related issues, compromising adequate implementation as well as monitoring and evaluation of national strategies [27, 35, 37, 46]. Simultaneously, the low qualification of professionals providing care to survivors of DV is influenced by negative socio-cultural factors and care providers demographic characteristics such as sex and age. Some studies reveal younger caregivers are less compassionate when screening for DV in their patients while older women show higher screening rates [47, 48]. These factors could compromise the and the non-judgmental environment to disclose DV events [2, 49] The lack of financial and human resources can also be substantiated by the fact that CSOs, where funding does not depend on general state funds, frequently design and disseminate DV prevention and control messages. Another example of the lack of government investment, is the existence of guidelines co-financed by a CSO. This is related to the management of sexual violence against children in almost all health facilities. Some studies have shown that although health sector financing is decentralised from state funds, the health sector did not plan a specific budget allocated to DV-related activities [50].

Besides the number of guidelines and protocols developed, it is crucial their content is aligned with recommendations of national and international policies advocating for comprehensive and adequate responses to DV which is not the current case. Furthermore, information included in these documents must be written and structured in such a way as to facilitate understanding and implementation of activities in all sectors involved [7, 14].

In general, the national legal documents have some limitations that may hamper their implementation. A few are focused on primary prevention, while most of them are focused on secondary prevention, compromising in some way the control over the occurrence of new violent episodes [24, 51, 52]. This can, to some extent, support the fact that most of the documents found in care services are aimed at assisting survivors of DV (care guidelines / protocols) and there were no documents available aimed at raising awareness for the prevention of DV (such as brochures, pamphlets). Although care for survivors of violence is advocated, little is done to operationalise the needed care and provide outreach to violence survivors. Another limitation is the lack of strategies for monitoring and evaluating DV prevention and control activities in the national legal documents, which could be one of the causes of the gap found in the translation of policies into practice [24]. There is a noticeable divergence regarding the content of documents under review. This may on one hand be justified by the specificity of the issues addressed in the guidelines, protocols, pamphlets and brochures. Part of this material has been designed to guide assistance or prevention activities, without other institutions’ involvement, thus compromising the multisectoral approach. This fact institutionalises the strategies for the prevention and control of DV, dispersing efforts, and compromising the rationalisation of resource use [20, 27, 53–55].

### Relevance of guidelines and protocols

It is known that government has developed laws and policies to fight against DV and the recognition of the relevance of these legal frameworks by providers is a demonstration of the commitment to combat and prevent DV. Nevertheless, the gaps in the development of guidelines and care protocols for DV survivors were manifested. As the WHO recommends for comprehensive and adequate care to DV survivors, consistency at the various levels of action is crucial, specifically at the state (the central level), the care providers (which includes not only the infrastructure but also the existence of qualified human resources to provide care and the users of these services, in this case the survivors [43]. This has also been described in some studies, where government changes have increased the political will to address DV from the top down to the care provider level. This reinforces the integrated and standardised care provision by ensuring advocacy, implementation, monitoring and evaluation of these instruments [56–58].

### Guidelines and protocols gaps and constrains

Responsiveness of the legal framework for DV – not only in terms of protecting the victim and punishing the aggressor – it also ensures responses are integrated which is crucial. Several factors have been described as key to the successful implementation of guidelines and protocols for addressing survivors of violence [59–62].

Some studies describe the lack of compatibility between the offense and the lightness of punishment given to the aggressor during judgement. This highlights the urgent need to conduct reforms in legislation [61, 63]. However, there is a recognition that enforcement of legislation is sometimes difficult. In short, DV survivors lack information on existing legal proceedings, and generally speaking, there is a palpable unwillingness to penalise offenders. On the other hand, fear of imprisonment of the perpetrators, high economic dependence of the survivors and socio-cultural attitudes of patriarchal dominance are factors likely to be related to the survivors’ failure to report on the event of violence and to the suspension of the judicial process [30, 63–69].

Given that DV is a multi-causal phenomenon, it is critical legislation reforms accommodate the contributions not only of the policy implementers, but also of DV survivors and communities in general. In order to ensure this, comprehensive and adequate interventions must be provided and accepted. CSOs should be included in this integrated approach, as these are often the gateway to the DV care provision system, given its connectivity with communities [7, 70]. In addition to community education and advocacy, CSOs also provide safety of DV survivors and their children by providing shelters [71, 72].

Therefore, it is essential, that care provided by these organisations can be detailed in a specific field of the standardised form recommended by the multisectoral mechanism for assisting survivors of violence in Mozambique. Herewith any other caregiver will be able to understand what assistance has been given to the victim at the CSO level. The inclusion of this field could encourage the continuity of care provision activities related to improving monitoring of the multisectoral mechanism [24, 73].

Another constraint is that each institution has its own DV case registration book, resulting in institutional databases. This can compromise the compilation of data on a single basis, contradicting what is recommended for the multisectoral approach and consequently the design and implementation of adequate DV prevention strategies based on evidence [24, 74].

Adequate implementation of policies and strategies for the prevention and control of DV, implies reasonable and adequately trained human resources. The lack of training of professionals is an obstacle compromising not only the understanding and applicability of policies and legislation on DV, but also the follow-up of DV cases. The poor performance of care service providers, was noticeable when questioning the events of violence and the willingness of the survivor to access other services which are part of the integrated care service [3, 75, 76]. Given the context of Mozambique and many other African countries, the influence of negative socio-cultural factors on the attitudes and practices of implementers regarding care provision for DV survivors it is not an unusual fact in this context. These factors function as barriers to develop help seeking behaviour which may increase DV survivors internalisation or anticipation of stigma [77, 78].

The facts described above were used by Gilson and Walt to develop a model of a policy analysis. In this model, they suggest incorporation of the policies context, content and process. Context refers to the environment in which the policy to be analyzed is inserted. Therefore, this process is a step-by-step policies design, implementation and if necessary, its reform. In addition to the components previously described, the authors recognise that a great deal of emphasis is being placed on the content of policies neglecting the role of the policy makers or implementers considered indispensable for the success of this model [41, 79].

This study has raised some relevant aspects in the design, and implementation of the guidelines/protocols. However, the lack of awareness of some focal points regarding the availability of documents in the study sites was noticeable. Therefore, it was difficult to make appropriate link between the awareness of individuals that were focal points and implementation of expected policies and practices.

## Conclusions

Although government commitment to mitigate DV is noteworthy given the amount of national policies, laws and strategic plans, guidelines and protocols produced. However, there is a dearth of guidelines, protocols, pamphlets and brochures in places providing care for survivors of violence. This makes it difficult to standardise and integrate these services. In addition, there was a misalignment between guidelines and protocols of attendance with policies, laws and strategic plans regarding their content and terminology used in their titles.

One of the strengths to promote DV control and prevention is recognition of the relevance of guidelines and protocols by gender focal points. However, they stress gaps compromising its proper implementation, so care delivery is adequate and timely. These facts lead us to recognise the lack of prioritisation in the governmental agenda and reinforce the need to monitor and evaluate the policies, laws, and strategic plans. This study also recognises the need to reform existing legislation, coupled with allocating an appropriate budget to ensure a proper strategies implementation and improving the survivors’ quality of life.

## Data Availability

Datasets used and/or analysed during this study are available from the corresponding author upon reasonable request.

## Abbreviations

DV: Domestic Violence
WHO: World Health Organisation
IMASIDA: Inquérito de Indicadores de Imunização, Malária e HIV/SIDA em Moçambique ((*Survey of the Indicators for Immunization, Malaria and HIV/AIDS in Mozambique*)
CSO: Civil Society Organisation
PAHO: Pan-American Health Organisation
UN: United Nations

## Footnotes

1 Mozambican Laws and Policies **Laws**: Constitution of the Republic (1990); Criminal Code (2014); Family Law (2004); Law on Domestic Violence against Women (2009); Legislation on the Promotion of Protection of the Child’s Rights (2009). **Policies**: Gender Policy and Strategy for its Implementation (2006). **Action/ Strategic plans**: Government Five-Year Plan (2015); National Plan of Action for the Prevention and Combat of Violence against Women (2007); Health Ministry Strategic Plan (2014); Police of the Republic of Mozambique Strategic Plan (2012); Strategic Plan for Institutional Development of the Ministry of Home Affairs (2014) Program of care for women and children - department of family and children victims of violence (2008); Intersectoral Mechanism of Integrated Attendance of Women Victim of Violence (2012); National Action Plan for the Advancement of Women 2010–2014 (2010); National Strategy for Basic Social Security (2016).

2 Ministry of Gender Child and Social Action – Responsible for the multisectoral national coordination of all gender-related activities, ensuring the provision of support services for victims of violence and monitoring & evaluation of the Multisectoral Mechanism for Integrated Care for Women Victims of Violence; Ministry of Health – Responsible for ensuring the access to health care for victims of violence; Ministry of Home Affairs – Responsible for assistance and protection to victims of violence and also for participating in the process of aggressor criminalization; Provincial Directorate of Gender, Social Action of Zambézia – Subordinated to the Ministry of Gender, Child and Social Action, which coordinates the activities ministry related at the provincial level; Provincial Directorate of Health of Zambézia- Subordinated to the Ministry of Health which coordinates health related activities at the provincial level.

3 Defined by the new law for prevention and combat premature unions, as union between people in whom at least one is a child (under the age of 18), formed with the immediate or future purpose of starting a family [42]
